# Door-to-door recruitment during the COVID-19 pandemic: Lessons learned from a population-based, longitudinal cohort study in North Carolina, USA

**DOI:** 10.21203/rs.3.rs-1234834/v1

**Published:** 2022-02-17

**Authors:** Jaclyn Karasik

**Keywords:** epidemiology, prospective cohort, recruitment methods, SARS-CoV-2, seroprevalence

## Abstract

**Background:**

The COVID-19 pandemic has disproportionately impacted many of the same communities that have been historically harmed by or underrepresented in public health research. In a prospective cohort study on COVID-19 in North Carolina, USA, we employed a door-to-door recruitment strategy on a randomly selected sample of households to maximize inclusivity and improve study diversity.

**Discussion:**

Rapidly shifting current events and an evolving pandemic required continuous updating of our approach. Using a variety of recruitment strategies and participation methods allowed us to quickly adapt and to reach a broad range of people with diverse needs and circumstances. Door-to-door recruitment had many unexpected benefits, allowing us to easily reach people that were working from home and leaving people with positive impressions of public health research. However, even when we were able to elicit a response from prospective participants, follow-up has remained a substantial challenge.

**Conclusions:**

It is paramount for public health practitioners to employ creative strategies and to invest time and resources to include hard-to-reach communities in research.

## Background

In the United States, the COVID-19 pandemic has had a disproportionate impact on socio-economically disadvantaged and rural communities, which suffer from longstanding health disparities and limited access to care (1). Therefore, it is critical to ensure that these individuals are represented in epidemiological studies and clinical trials, despite the fact that these communities have been historically under-represented in public health research (2, 3).

We conducted a population-based, longitudinal cohort study to estimate incidence and community prevalence of SARS-CoV-2 infections in Chatham County, North Carolina (4). Initial recruitment efforts included 1536 addresses selected in a stratified, two-stage probability-based sampling design. All sampled households received postcards or emails; those with phone numbers who did not respond to mail were given phone calls. Door-to-door home visits were attempted among a subset of those who still did not respond to postcards or phone calls.

Despite oversampling in regions of the county with lower median income levels and/or higher concentrations of Hispanic/Latino and Black/African American populations, interim analysis of our participants after five months of postcard and phone recruitment suggested that our cohort did not reflect the demographics of Chatham County (4). In brief, the cohort was skewing toward older, wealthier, and predominantly White participants. We hypothesized that our recruitment efforts, initially limited by concerns regarding shortages of personal protective equipment and restrictions on face-to-face interactions, would not yield a representative cohort due to differential nonresponse across population subgroups (5, 6). As those concerns subsided, we implemented a door-to-door recruitment approach in February 2021 to increase representation within the cohort to better reflect the underlying population. In this paper, we share challenges and lessons we have learned along the way while recruiting for this study.

## Lesson 1: Current Events Affected Recruitment

Monitoring current events was key to anticipating and successfully navigating barriers to study recruitment. Initial recruitment efforts benefited from contacting sampled individuals at a time when there was a widespread feeling of powerlessness. Individuals frequently perceived participation in the study as an opportunity to “help fight COVID-19.” However, we observed a clear decrease in response to mailings and phone calls during the lead up to the 2020 United States presidential election. This was a time when many residents were already receiving high volumes of election-related mail and phone calls. After this subsided, enthusiasm continued to wane with the rapid roll-out of vaccines and the onset of “pandemic fatigue” (7).

Some challenges remained consistent throughout the recruitment period. Political ideology affected how people adapted COVID-19 preventative behaviors and mistrust in public health authorities remained widespread (8,9). Recruitment attempts by study staff were frequently met with skepticism and even dismissiveness from individuals choosing not to adhere to preventative measures such as masking or social distancing.

In addition, it was difficult to engage local organizations in recruitment efforts as many were already stretched thin while responding to a surge in community need, including food and economic insecurity. Asking staff to assist with door-to-door recruitment would have placed additional burden on these organizations already responding to a crisis. Community organizations were able to provide some consultation in the development of the study, and we were able to provide gift cards to support their work.

## Lesson 2: Recruitment Methods Changed With Greater Understanding Of Transmission Modes

Knowledge of SARS-CoV-2 transmission modes and dynamics were still uncertain when study recruitment efforts began in July 2020. Community engagement is a critical aspect of public health research (10), but in-person interactions at local community centers and churches were initially limited due to lockdowns and concerns for study staff safety. Once the team began door-to-door recruitment, concerns regarding spread via surfaces (e.g., tablets, pencils, etc.) and close contact during conversations necessitated meticulously designed and rehearsed door-to-door recruitment protocols. For example, we began by only collecting contact information of interested participants in order to limit face-to-face contact time while visiting homes, even as most encounters were conducted outside and at distance of at least six feet. Unfortunately, the limited information often led to attrition when staff attempted to follow up at a later time to complete the consent process by phone. Once study staff were vaccinated and more evidence regarding the limited potential of outdoor transmission emerged, we found that being able to complete the full consent process at their homes helped to secure enrollment. In short, our recruitment methods needed to adapt to the changing state-of-knowledge.

## Lesson 3: Diverse Methods Yielded Diverse Participants

Chatham County encompasses both rural and semi-urban towns, a large Spanish-speaking and African American population, and populations of highly variable socioeconomic status and age ranges, including working families to retirees. The county also spans a large geographic area (18th largest out of 100 counties in North Carolina). Employing diverse recruitment methods meant that we reached out to people through email, postal mail, phone calls, and door-to-door visits to homes. This was especially important during the COVID-19 pandemic when the digital divide caused additional barriers to resources for many (11). We also offered diverse ways to participate in the study to maximize inclusivity and offset concerns about transmission risk in public spaces. We offered to collect blood and nasal swab samples at an established health clinic in the vicinity of their home (up to 20 minutes away by car) or allowed participants to self-collect samples using at-home sampling devices for both blood and nasal swabs. Similarly, we offered online and written mail-in surveys for those without internet or email. Thus, those who were not comfortable going out in public due to the threat of COVID-19 or lack of transportation were still able to participate without leaving their homes.

In addition, diversity in our field staff (African American, White, and Spanish-speaking/Latinx) reflected demographics of Chatham County and helped to build trust with potential participants. We also found that introducing ourselves as students was a disarming icebreaker that opened up the conversation enough for us to explain the purpose for our visit. We found that Spanish-speaking potential participants often became much more interested in learning about our work once they realized that a field staff member spoke Spanish. Research staff also worked to raise awareness about the study more broadly in the community by handing out bilingual flyers at a holiday market and working with community partners. In brief, employing a range of recruitment methods, offering multiple avenues to participate in the study, and having a diverse team of field staff helped to support recruitment of diverse participants that reflected the demography of Chatham.

## Lesson 4: Follow-up Remained A Challenge

In addition to challenges with enrollment, it was also difficult to keep participants retained and engaged at all phases of the study. Several individuals consented to participate but never made it to their first clinic visit. While our monthly clinic visits only lasted about 15 minutes, our clinics are held on weekdays from 8am–5pm, which posed challenges for those with less freedom to take time off work. We offered mail-in test kits to attempt to address this issue, but many participants still had difficulties making time for the study.

An unanticipated challenge in recruiting participants involved cultural considerations. Several households offered their contact information out of what we now perceive as courtesy or politeness but then were difficult to reach to complete the enrollment process. As a result, we switched to enrolling participants while visiting their homes to reduce attrition. We also emphasized that the study was voluntary, and they were not obligated to give their contact information if uninterested. Enacting this new strategy resulted in consenting 11 participants on the spot.

**Figure 1:**
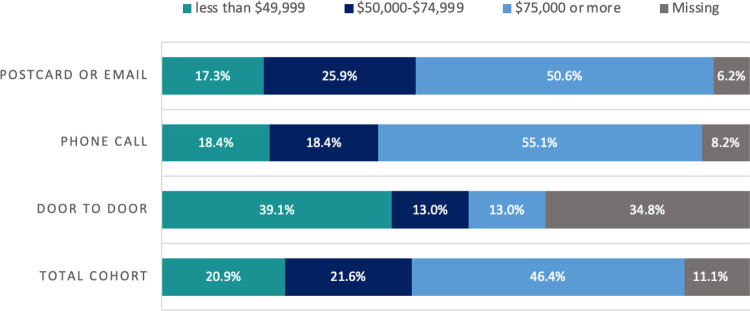
Income categories of participants in Chatham County, NC COVID-19 Cohort (n=153), stratified by method of recruitment

**Figure 2:**
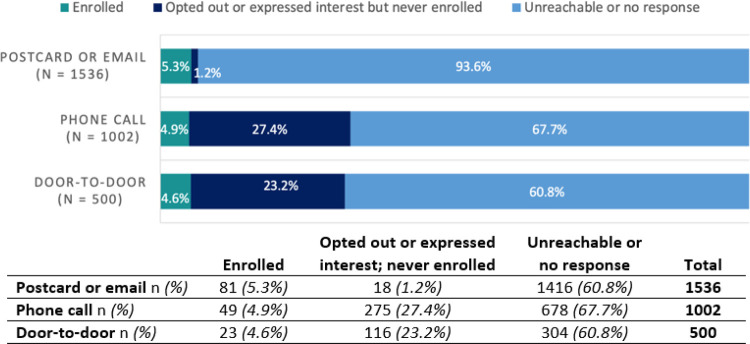
Chatham County COVID-19 Cohort Study Recruitment Outcomes (sample size of n=1536) All eligible households in the sample were contacted via postcard or email. Households with available phone numbers were then given phone calls. Households that had still failed to respond were contacted via door-to-door outreach; not all non-responsive households were contacted due to resource limitations.

Door-to-door successfully yielded participants that were younger, had a lower income, and were more diverse in terms of race and ethnicity in comparison to participants recruited through other methods (Table 1; Figure 1). This was expected because we targeted our door-to-door recruitment geographically to improve reach of persons with these demographics. It also resulted in similar enrollment yield compared with other methods (Figure 2), with 4.6% (n=23) of homes visited enrolling in the study. However, door-to-door efforts did not yield enough participants to significantly increase the size or overall diversity of our cohort by race, ethnicity, or age. We anticipated this difficulty with enrolling homes in the door-to-door recruitment stage since they had already declined to respond to mailings and phone calls.

Unfortunately, we also have seen the greatest rate of withdrawal among participants recruited with the door-to-door method compared to others. Many of these participants missed initial visits and study staff put in extra effort to confirm appointments with participants to ensure the best chance of adherence. As of November 2021, about 43% of door-to-door participants have withdrawn compared to 16% of participants recruited by phone and 17% of postcard/email participants. We expected this as many of the door-to-door participants are socio-economically disadvantaged and likely to face additional barriers to participation and retention in research, compared to participants from socio-economically advantaged groups (12). Some barriers identified in previous reviews include mistrust in research and the medical system, limited transportation, time and financial constraints, and family obligations (13,14).

## Lesson 5: In-person Recruitment Had Unexpected Benefits

With many people spending more time at home during the pandemic, it was often more accessible to reach participants at home on the weekdays in addition to weekends. Even when households were not interested in participating, they frequently expressed a sense of appreciation for our study and for the time spent traveling to their home. For example, one household shared their reservations about the COVID-19 vaccines and the source of the pandemic, but still positively commented on our efforts to serve Chatham County and asked thoughtful questions about our work. Thus, even when households did not participate, our encounters provided Chatham County residents with the opportunity to develop a positive perception and awareness of research efforts. Ideally, this could increase willingness to participate in future studies.

## Conclusion

Conducting a longitudinal, population-based cohort study of a highly transmissible respiratory virus in the midst of global pandemic presented many challenges, especially when attempting to recruit and retain a representative cohort including diverse populations. Our first-hand experience highlighted how secular events may influence possible participants towards or against research. Similarly, our enrollment patterns demonstrate how recruitment strategies must be responsive to the needs of the target population. No single method can achieve adequate representation in population-based studies, and multi-mode data collection allows for recruitment of a more diverse population. In conclusion, lessons learned from our ongoing study will inform future community-based research during quickly evolving public health threats.

## Figures and Tables

**Table 1.** All demographic information was collected prior to the participant’s first visit or sample collection via email or paper surveys for participants without email.

	Postcard or Email *(n=81 enrolled participants)*	Phone Call *(n=49)*	Door to Door *(n=23)*	All participants *(n=153)*
**White Non-Hispanic *n / total responses (%)***	62/77 *(80.5%)*	38/43 *(88.3%)*	6/15 *(40%)*	**106/135 *(78.5%)***
**Median Years of Age *(Q1, Q3)***	63 *(51, 70)*	60 *(47, 67)*	45 *(35, 59)*	**61 *(47, 68)***
**Income *n (%)***				
**less than**	14 *(17.3%)*	9 *(18.4%)*	9 *(39.1%)*	**32 *(20.9%)***
**$49,999**	21 *(25.9%)*	9 *(18.4%)*	3 *(13.0%)*	**33 *(21.6%)***
**$50,000-$74,999**	41 *(25.9%)*	27 *(55.1%)*	3 *(13.0%)*	**71 *(46.4%)***
**$75,000 or more**	5 *(6.2%)*	4 *(8.2%)*	8 *(34.8%)*	**17 *(11.1%)***
**Missing**				

## Data Availability

The datasets used and analyzed during the current study are available from the corresponding author on reasonable request.

## Abbreviations

COVID-19: coronavirus disease 2019
SARS-CoV-2: severe acute respiratory syndrome coronavirus 2
